# Sulfamethoxazole Removal from Drinking Water by Activated Carbon: Kinetics and Diffusion Process

**DOI:** 10.3390/molecules25204656

**Published:** 2020-10-13

**Authors:** Mohamed BIZI

**Affiliations:** BRGM, Water, Environment, Processes Development & Analysis Division 3, Avenue C. Guillemin, 45060 Orleans, CEDEX 2, France; m.bizi@brgm.fr; Tel.: +33-(0)2-38-64-36-62

**Keywords:** activated carbon, diffusion, kinetic, micropollutants, pharmaceuticals, sulfamethoxazole, water treatment, wastewater

## Abstract

Sulfamethoxazole (SMX), a pharmaceutical residue, which is persistent and mobile in soils, shows low biodegradability, and is frequently found in the different aquatic compartments, can be found at very low concentrations in water intended for human consumption. In conditions compatible with industrial practices, the kinetic reactivity and performance of tap water purification using activated carbon powder (ACP) are examined here using two extreme mass ratios of SMX to ACP: 2 µg/L and 2 mg/L of SMX for only 10 mg/L of ACP. In response to surface chemistry, ACP texture and the intrinsic properties of SMX in water at a pH of 8.1, four kinetic models, and two monosolute equilibrium models showed a total purification of the 2 µg/L of SMX, the presence of energetic heterogeneity of surface adsorption of ACP, rapid kinetics compatible with the residence times of industrial water treatment processes, and kinetics affected by intraparticle diffusion. The adsorption mechanisms proposed are physical mechanisms based mainly on π–π dispersion interactions and electrostatic interactions by SMX^−^/Divalent cation/ArO^−^ and SMX^−^/Divalent cation/ArCOO^−^ bridging. Adsorption in tap water, also an innovative element of this study, shows that ACP is very efficient for the purification of very slightly polluted water.

## 1. Introduction

Sulfamethoxazole (SMX), an antibiotic for human and veterinary use, figures among the most commonly found residues in different aquatic compartments [1,2]. After being consumed, this antibiotic is metabolized in proportions not exceeding 70% [3]. A non-negligible quantity of this active ingredient and its metabolites is therefore excreted, mainly in urine, and enters wastewater in urban areas, or is directly released into the environment in the case of livestock farms. Its concentration can range from 10 to 1500 ng/L in wastewater treatment plant (WWTP) effluents, from 0.2 to 1100 ng/L in groundwater, and from around 100 pg/L to a few ng/L in water intended for human consumption [4,5,6,7,8]. Heather E. Gall et al. [8] found 26 groundwater sites in the US (West Branch of the Susquehanna River basin) with SMX concentrations ranging between 0.1 and 32 µg/L, with an average of 17.1 µg/L. It was among the most prevalent antimicrobial contaminants detected in a nationwide groundwater survey conducted by the United States Geological Survey [9].

Wastewater treatment plants (WWTPs) have been identified as the main channel through which pharmaceutical residues are introduced into the natural environment. They were not designed to treat micropollutants. The reduction rate is highly variable within a single plant and from one plant to another. WWTPs are however capable of eliminating a large share of the substances present in the inputs. The reduction rate of SMX varies between 50 and 74% [2,10,11].

Although necessary for curative and preventive purposes, this molecule can have an unintended effect on non-target organisms present in the environment. The release of SMX, and more widely, antibiotics in general, into the environment can lead to the emergence of resistant bacterial strains [12]. Antibiotics can also (i) disrupt the biological treatment process implemented in WWTPs by damaging the microorganisms present in activated sludge (biological treatment), and (ii) lead to potential toxicity for aquatic organisms [2,13,14]. These toxic effects may be reinforced in the presence of other antibiotics [15]. Furthermore, SMX can accumulate in certain plants (wheat, tomatoes, spinach) [16]. The physicochemical properties of this molecule mean that it is difficult to reduce its presence in soils. SMX is an amphoteric, water soluble compound with a low pKa and carries a negative charge in alkaline water (WWTP and surface waters), with low biodegradability, making it persistent and mobile in soils. At alkaline pH, electrostatic repulsion occurs between this molecule and the main components of natural soil (minerals and organic matter).

Effective, economical, and sustainable measures suited to current industrial water treatment processes and which do not require major investments should be designed and developed to remove micropollutants in order to facilitate the safe release and potential reuse of wastewater and to produce good quality water for human consumption. Many technologies exist to treat pharmaceutical residues; however, their implementation in different types of treatment processes for wastewater and water intended for human consumption is balanced against their cost and sometimes faces technical difficulties: poor compatibility with existing facilities, reliability of facilities, production of by-products, or degradation metabolites, etc. In terms of the removal of this pharmaceutical contaminant, numerous physical and chemical techniques and methods—such as adsorption, degradation by UV radiation, photochemical degradation, degradation by ozonation, Fenton oxidation, biodegradation and membrane filtration—have been assessed [17,18,19,20,21,22,23]. The adsorption technique was proven using a good adsorbent, the treatment method of choice due to its high efficiency, its low cost, and its simple functioning. Furthermore, adsorption is one of the treatment methods used for water intended for human consumption and is indirectly used for primary treatment in WWTPs (adsorption on sediments transported by wastewater). This technology is often suggested for the treatment of slightly polluted water. Different materials, such as clay, zeolite, carbon nanotubes, graphene oxide, nanocomposites, and TiO_2_ nanoparticles, have been examined for their removal efficiency of SMX from aqueous solutions [24,25,26]. Typically, these studies are done in milli-Q water and at high concentrations of SMX. It is worth highlighting that the literature does not currently offer any data on the treatment of SMX at very low concentration in drinking water. For health and environmental reasons, it is neither advisable nor conceivable to use nanoparticles (spheres, nanotubes, nanowires, or with another shape) to remove organic or inorganic micropollutants. The release of such materials into the environment could be detrimental to human health and to the ecological biosphere. Titanium oxide for instance, whether nanometric or micrometric, is classified as a possible carcinogen for humans and animals [27,28]. Based on bibliographical and industrial data, activated carbon stands out as a suitable material for the removal of pharmaceutical micropollutants [29,30]. Activated carbons can be distinguished by their rather specific texture and surface chemistry. They are generally highly microporous with high specific surface areas. Irregularities in their microcrystalline structure and the presence of heteroatoms (oxygen, hydrogen, etc.) lead to the formation of functional groups at their surface, the nature of which depends mainly on the processes used for their activation [31,32,33]. These functional groups (adsorption sites) give activated carbon acido-basic properties.

We therefore propose to examine the performance of an industrial activated carbon powder (ACP, Norit SA Super) for SMX removal as a final phase in drinking water treatment as well as for application in tertiary treatment in a WWTP. The aim of this study is (1) to examine the efficiency of SMX removal from drinking water in conditions compatible with the analytical observations carried out in groundwater and in water intended for human consumption and also with industrial requirements (low-cost)—very low SMX concentrations (2 µg/L) and low concentration of solids; (2) to define ACP behavior and kinetic adsorption mechanisms in the case of a very low concentration of this compound; and (3) to identify the functional groups involved in SMX/activated carbon interactions.

## 2. Results and Discussion

### 2.1. SMX Characterization

**Figure 1:**
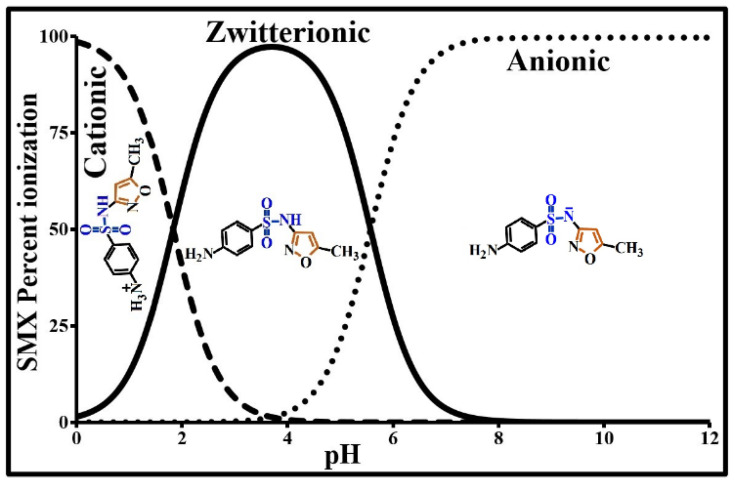
Sulfamethoxazole (SMX) speciation as a function of pH in aqueous solution.

SMX is a polar, UV-sensitive molecule composed of a sulfonyl group located between an amine group and an aniline group. It has amphoteric properties with acido-basic characteristics. More specifically, it contains a basic amine group (-NH_2_) and an acidic amide group (-NH-). The amine group is able to accept a proton, while the amide group is able to donate a proton in specific pH conditions (Figure 1). Its speciation in pure water as a function of pH and of its dissociation constants, established based on the Henderson–Hasselbalch method and Equation (1), shows that this molecule is zwitterionic between its two pK_a_ values, practically cationic below pK_a1_ and anionic above pK_a2_. It is completely anionic above pH 7.5 (Figure 1). Its solubility depends, among other factors, on the pH, temperature, and ionic strength of the carrier medium. It is minimal at its isoelectric point (IEP = 4) and, for a given pH, it increases with temperature. On the basis of the experimental data of Zhang et al. [34], the solubility of SMX in pure water below 60 °C can be expressed by the empirical function given below Equation (2). The electronic absorption spectrum of this molecule is characterized by several absorption bands all located in the UV band with varying intensities and positions according to pH. The optical density of the most intense absorption band due to π→π* electronic transition was used, via a calibration, to determine the quantities of SMX adsorbed by the activated carbon based on the remainder method. In an alkaline medium, this transition is situated at 257 nm.

(1) $$PI=\frac{100}{1+10^{x\left(pH-pK_{a}\right)}}$$

where *x* = −1 and *pKa* = *pK*_*a*1_ if an acid drug is used or *x* = 1 and *pKa* = *pK*_*a*2_ if a basic drug is used.

(2) $$S=aT+bT^{c}$$

where *a* = 14.80, *b* = 4.63 × 10^−6^, *c* = 4.43, *T* in °C (<60 °C), and the solubility *S* in mg/L.

### 2.2. Activated Carbon Characteristics

**Figure 2:**
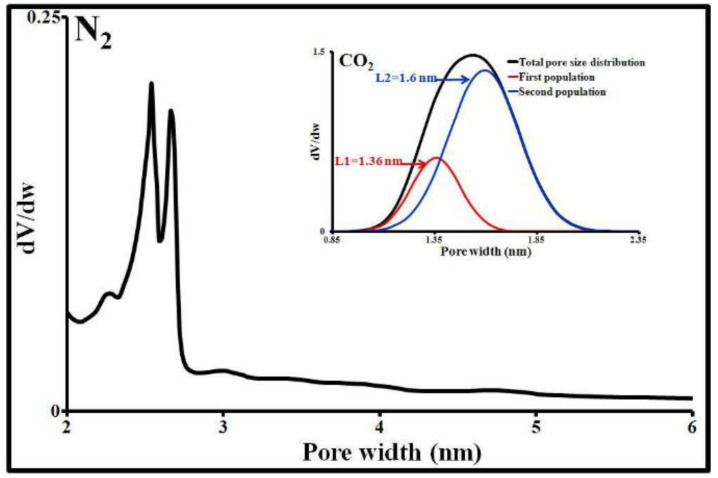
Pore size distribution obtained by N_2_ desorption at 77 K and CO_2_ adsorption at 273 K.

The textural properties of an adsorbent affect the adsorption equilibria. Knowledge of these properties is essential to more accurately interpret the adsorption mechanisms of all molecules, whether organic or otherwise. The textural analysis of the ACP was therefore performed by N_2_ adsorption/desorption, CO_2_ adsorption, and by particle size measurement. Its main texture parameters are provided in Table 1. This activated carbon has a particle size of less than 120 µm and an average diameter of around 24 ± 1 µm. 7.5% by mass of its particle size distribution is below 2 µm and 56% is below 20 µm. It is also characterized by a large specific surface area, as well as a polydisperse pore size distribution, with 95% below 3 nm and 64% below 2 nm (maximum micropore size). Virtually all of its mesopores are between 2 and 6 nm. The macropore population (>50 nm) is negligible (Figure 2). These meso- and macropores contribute to SMX transport by diffusion of the liquid phase into each particle. The minimum micropore size for this activated carbon (0.95 nm) is accessible to SMX (width and height <0.6 nm). The specific surface areas of its micropores, mesopores, and macropores represent respectively 73%, 21%, and 6% of the total specific surface area according to the t-plot model. N_2_ and CO_2_ give virtually the same micropore surface areas and volumes.

Activated carbons have surface functional groups, the nature of which is dependent on the origin and chemical composition of their raw material as well as their preparation and activation method. These functional groups can be considered acidic or basic sites promoting ion adsorption. The most commonly found acidic sites at the surface of activated carbon are carboxyl (Ar-COOH), phenol (Ar-OH), carbonyl (Ar-C=O), anhydride (Ar-(C=O-O-O=C), ether (Ar(-O-)Ar’), quinone (O=Ar=O), and lactone groups (Ar-C=OO-Ar’). Basic sites can be associated with two types of structures: (i) chromene and pyrene; and (ii) Lewis structures associated with π electron-rich regions situated on basal planes [31,32,33]. Furthermore, part of the basicity could also be attributed to the intrinsic properties of the ash [35]. The identification and quantities of these functional groups, determined using the Boehm method, are provided in Table 2.

The sites revealed by this quantification method are present in very significant proportions, with the exception of lactones. The phenol and carbonyl densities are almost 2 to 3 times higher than those of carboxyls and anhydrides. These functions make the carbon surface more polar and increase its affinity for water through the formation of hydrogen bonds. There are slightly more electron donors than acceptors. The concentrations of acidic and basic sites are 1.5 and 1.3 µmol/m^2^, respectively. The ratio of the concentrations tends towards neutral behavior, confirmed by the PZC value of 6.8 for this activated carbon. Above the PZC, the phenol and carboxyl groups dissociate by releasing their proton (H^+^) and obtaining a negative charge. Below this pH, the activated carbon’s overall surface charge becomes positive and can be attributed to the basic functional groups. At tap water pH, the activated carbon Norit SA Super is negative and SMX is also negative. If we exclude the cations present in tap water, the electrostatic interaction between the adsorbent and the adsorbate is completely repulsive.

### 2.3. Equilibrium Isotherm and Adsorption Mechanism

**Figure 3:**
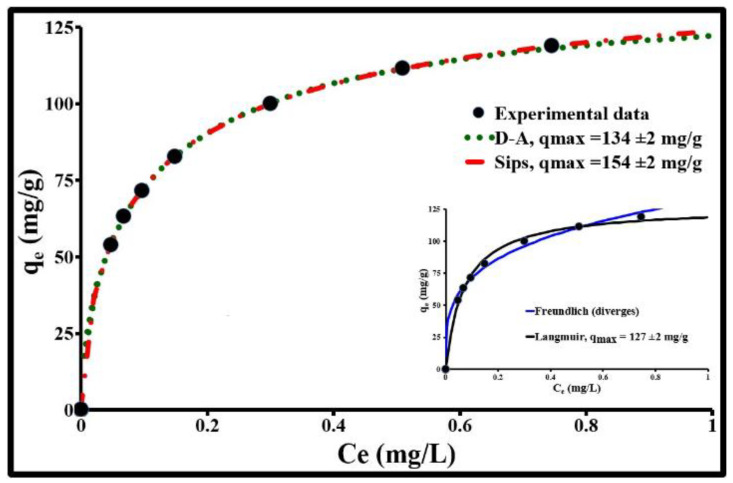
SMX adsorption isotherm on ACP Norit SA Super at pH 8.1 in tap water solution.

The ACP dose required for the total adsorption of 2 µg/L of SMX was found by evaluating the adsorption capacity of 5 doses of ACP: 3, 6, 8, 10, and 12 mg/L. Their respective adsorption rates are 80, 97, 99, 100, and 100%. At 10 mg/l, the removal efficiency becomes almost constant. Therefore, this concentration corresponds to a very favorable optimum. In the applied experimental method (10 mg/L of ACP; 2µg/L to 2 mg/L of SMX), the isotherm for SMX adsorption by ACP (Figure 3) is H2 according to Giles’ classification of adsorption isotherms [36]. Its shape indicates a very significant affinity between the adsorbent and the adsorbate in tap water (pH 8.1). As an initial approximation, this isotherm can be described by three distinct regions: (1) a region with relatively high adsorption energy represented by a vertical slope of the isotherm representing the reaction of the most active available sites when placing a low concentration of SMX in solution. The maximum amplitude of this adsorption is approximately 0.2 mg/g. (2) An increasing adsorption region represented by a curved isotherm. The isotherm shape in this location characterizes the presence of zones with heterogeneous surface energies, where the most important rupture with the 3rd phase is located at a concentration, at equilibrium *C_e_*, of 0.51 mg/L and adsorption of 111.6 mg/L. (3) A region of very slow adsorption leading towards a plateau which finalizes the monolayer adsorption.

The SMX equilibrium isotherm was analyzed by Freundlich, Langmuir, Dubinin–Astakhov (D–A), and Sips isotherm models using a non-linear regression technique. As shown in Figure 3, the first two models do not fit the experimental data correctly. Their relative standard deviations (∆q) from the experimental data are 4 and 3%, respectively. Furthermore, the Freundlich model diverges and the Langmuir model does not take into account the surface energetic heterogeneity of the activated carbon. The D–A model and the Sips model perfectly fit the experimental data; the coefficient of determination “R^2^” obtained in both cases is 0.9999 and the relative deviation between the experimental values and the calculated values is 0.4%. The maximum quantity of SMX “q_max_” that can be adsorbed according to these two models is between 134 and 154 mg/g, respectively. The macroscopic parameters describing this situation are n_D_ = 1.51 ± 0.04, E = 5.7 ± 0.1 kJ/mol, n_S_ = 0.66 ± 0.01, and K_S_ = 8.5 ± 0.3 L/mg. The values obtained for n_D_ and E clearly show that the surface energies involved in adsorption are heterogeneous (n_D_ > 1) and that physisorption is the dominant SMX adsorption method on the activated carbon Norit SA Super in tap water (E < 8 kJ/mol). n_S_ (<1) and K_S_ confirm this observation. The energetic heterogeneity is mainly associated with the different surface functions and the texture (pore shape and geometry, fractal, tortuosity, roughness, etc.) of the ACP. The narrowest pores (<2 nm) are sites with higher energy and are, therefore, those which will adsorb more SMX at lower concentrations. The adsorption capacity at these concentrations is, therefore, proportional to the microporous volume. The surface chemistry of the largest pores will have a strong influence on the highest concentrations. Intraparticle diffusion and adsorption will be greatly influenced by the geometric dimensions and orientation of SMX molecules, pore width, and the type of functional groups of the ACP. According to the SMX orientation, its projected surface area will be different and subsequently, its diffusion into the micropores will depend on it. Furthermore, on the basis of these dimensions, this molecule cannot enter micropores with an opening width “L” less than 1.5 to 2 times its height “h”: 1.5 ≤ L/h ≤ 2 [37]. The limit is, therefore, 0.9 nm, which perfectly matches the dimensions of PAC micropores determined by CO_2_ adsorption (Figure 2).

The adsorption activity of activated carbon in suspension in tap water (pH 8.1) is due to the nature of its functional groups, its textures, and the intrinsic properties of SMX in relation to the physicochemical characteristics of the medium. The presence of basic groups leads to an increase in the density of delocalized π electrons in the graphene layers and thus, an increase in the carbon’s adsorption potential, while the acidic groups accentuate the local density of π electrons in the graphene layers [38,39].

Given its composition and chemical structure, SMX^−^ is able to initiate (i) π–π stacking interactions between π electrons in its aromatic core and π electrons in the graphene [40,41,42,43], (ii) electrostatic interactions by bridging, (iii) the amine group of SMX^−^ can also react with the functional groups of the activated carbon containing oxygen such as carbonyl and phenol, and finally, (iv) the presence of hydrogen bonds is not impossible. All these physical bonds can occur simultaneously. The presence of Ca^2+^ and Mg^2+^ in low concentrations in tap water can be at the origin of electrostatic interactions between the activated carbon and SMX^−^ by producing bond bridges (-/+↭+/-): SMX^−^/Cation/ArO^−^ and SMX^-^/Cation/ArCOO^−^. In the suspensions used, these two cations are found at respective concentrations of 2.06 and 0.47 meq/L and have electrostatic and thermodynamic characteristics which promote these interactions. Their hydration free energy (∆G_solv_) is, respectively, −380.8 and −455.5 kcal/mol, their polarizability is 3.161 and 0.481 a.u, and their ionic potential is 2 and 2.78 [44]. Although the favored coordination for the Mg^2+^ ion is sixfold octahedral, Ca^2+^ shows a greater diversity of coordination numbers (5 to 8), with seven- and eightfold coordination the most common [45].

Ca^2+^ and Mg^2+^ ions prefer to bind to hard ligands containing oxygen and/or nitrogen, with a preference for oxygen (strong electronegativity). In the case of interaction, they will mainly form electrostatic interactions [46]. However, since the Mg^2+^ ion interacts strongly with six molecules of water in [Mg(H_2_O)_6_]^2+^, it is unlikely that SMX^−^, a larger anion, will easily replace water to give an SMX^−^/Mg^2+^ complex. On the other hand, the larger Ca^2+^ ion will bind more easily and strongly to SMX^−^, a larger anion than water; the final dipole will in turn build an electrostatic bond with the activated carbon.

### 2.4. Sorption Dynamics

#### 2.4.1. Initial Adsorption Rate

**Figure 4:**
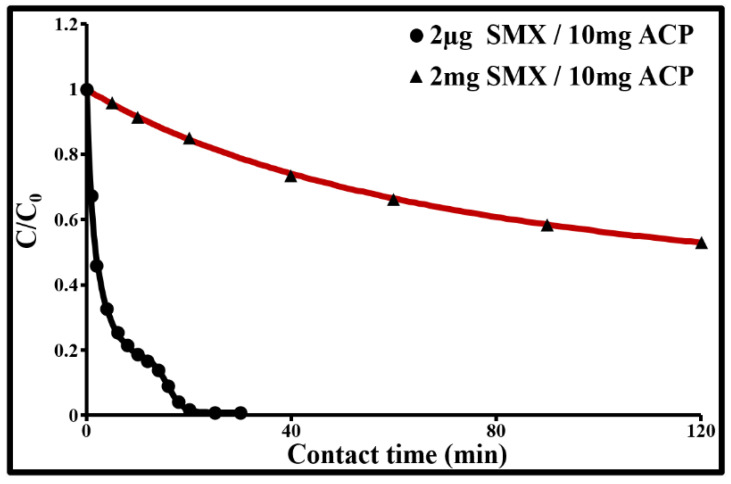
Dimensionless concentration as a function of time for SMX adsorption by ACP in drinking water at 293 K. (Values given at ± 2 ng/L for 2 µg SMX/10 mg ACP and at ± 0.03 mg/L for 10 mg SMX/10 mg ACP).

The initial SMX adsorption rate by 10 mg/L of ACP, determined from the slope of the initial straight line of the curve showing the evolution of the dimensionless ratio C/C_0_ in f(t), is dependent on the SMX concentration (Figure 4). It sharply decreases when the SMX concentration is increased from 2 µg/L to 2 mg/L. The adsorption rate constant is around 0.27 s^−1^ for 2 µg/L and 0.006 s^−1^ for 2 mg/L of SMX. The fact that the sorption rate increases when the initial SMX concentration is decreased suggests that the limiting mechanism in the kinetics involves transport phenomena in the ACP particles. At 2 µg/L, the C/C_0_ curve as a function of time has three inflection points which mark the transition between four transport mechanisms for SMX molecules. The sorption rate at 2 min reaches 54% for 2 µg/L and 1.5% for 2 mg/L. At 30 min, it reaches 100% and 21% for the two respective concentrations. Finally, for 2 mg/L, C/C_0_ stabilizes at 0.4 after 4 h of contact and at 0.39 ± 0.02 after 24 h. A high concentration acts as a driving force in the SMX diffusion and fixation processes.

#### 2.4.2. Sorption Diffusion Mechanisms

Sorption at the liquid/solid interface, with adsorption occurring locally in the pores, includes, in kinetic terms, three important stages:

- Diffusion through the film around the solid particles of adsorbate towards the outer surface, known as external diffusion (external mass transfer);
- Diffusion into the pores of the adsorbate, known as “intraparticle diffusion”;
- The adsorption/desorption reaction, i.e., the “surface reaction”.

**Figure 5:**
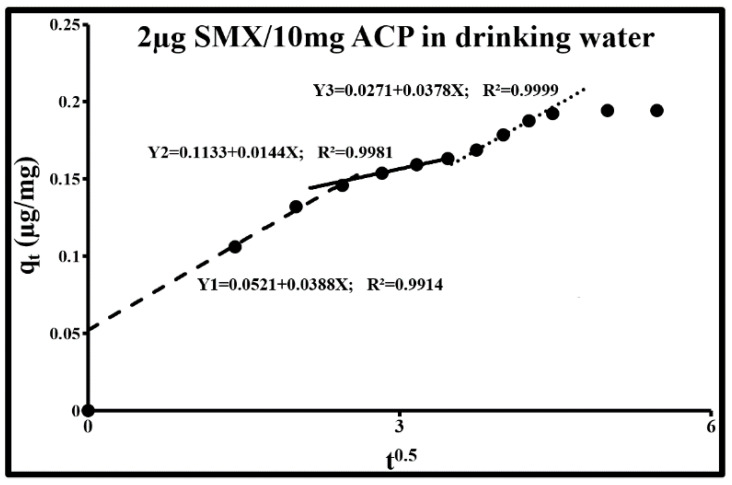
Weber–Morris plots for the sorption of SMX by ACP Norit SA Super in drinking water. (SMX: 2 µg/L; ACP: 10 mg/L; pH = 8.1, *T* = 293 K).

**Figure 6:**
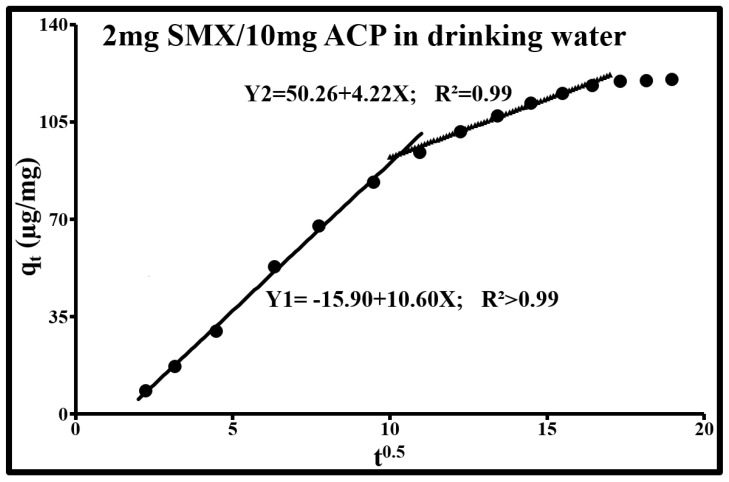
Weber–Morris plots for the sorption of SMX by ACP Norit SA Super in drinking water. (SMX: 2 mg/L; ACP: 10 mg/L; pH = 8.1, *T* = 293 K).

The study of these mechanisms in the case of SMX adsorption by ACP is based on the Weber and Morris kinetic intraparticle diffusion model, the pseudo-first-order kinetic model, the pseudo-second-order kinetic model, and the double exponential kinetic model. The Weber and Morris kinetic intraparticle diffusion model is used to determine the phenomenon that limits the sorption mechanism. According to this model, for the two SMX concentrations, sorption occurs in three dynamic phases with a continuity between external mass transfer and internal diffusion. These phases are revealed through a multilinear curve composed of several segments (Figure 5 and Figure 6; Table 3). The first phase consists of the limitation of adsorption by external diffusion (1r segment: Y1). The second consists of the gradual adsorption of the solute limited by intraparticle diffusion (2nd and 3rd segment for SMX at 2 µg/L; 2nd segment for SMX at 2 mg/L). The third phase corresponds to a state of equilibrium during which the adsorption capacity remains stable (plateau). Figure 5 and Figure 6 clearly show that the straight lines containing these segments do not pass through the origin. The slopes of these straight lines are equal to the ki diffusion constants. The values of the non-zero y-intercepts correspond to the “C_i_” constants given in Table 3. The existence of these constants fully supports the coexistence of external diffusion and intraparticle diffusion. A large deviation from the origin indicates that the diffusion of the boundary layer affects adsorption. These constants are directly related to the SMX concentration in the boundary layer and the thickness of this layer. The higher these values, the greater the effect of the boundary layer. All these mechanisms can be assimilated with a resistance in series of mass transfer of the adsorbate from the solution medium to active adsorption sites. During the global trajectory, the flow, the duration of each step, and the sorption rates vary according to the roughness, tortuosity, constrictivity, particle size, the physicochemical conditions of the medium, and the type of activated carbon functional groups (surface chemistry). Additionally, external diffusion depends on the hydrodynamic conditions of the medium.

At a SMX concentration of 2 µg/L and an ACP concentration of 10 mg/L (Figure 5), the three segments are attributed, respectively, to external diffusion, intraparticle diffusion in micropores, and intraparticle diffusion in mesopores. Their durations are approximately 6.3, 7.2, and 5.5 min. The final equilibrium phase is reached rapidly. The values of the C_i_ constants are proportional to the thickness of the boundary layers of the above-mentioned zones. The micropore constant C_2_ is equal to 2.2 × C_1_ and 4.2 × C_3_. The resistance of the micropore boundary layer is therefore higher and thus, led to a drop in diffusion rate (k_i2_ < k_i2_ and k_i2_ < k_i3_; k_i_ = Yi slope). In the micropores where the pore size is comparable to the size of adsorbate molecules, diffusion is highly restricted by the presence of pore walls. The sum of the C_i_ constants is exactly equal to the quantity of SMX completely adsorbed by the ACP, i.e., 0.2 mg/g. Its distribution between the external surface, the micropores, and the mesopores is, respectively, 26.1%, 56.7%, and 13.7%. The behavior of macropores is included in that of the external surface. In accordance with the geometry of these three zones, it is more appropriate to employ the term “boundary layer” for the external surface of particles and for mesopores; however, for micropores, it is a volume in suspension introduced into the micropores and whose thickness corresponds to the average deviation (or average diameter) of these micropores (micropore filling). SMX diffusion, therefore, occurs in this volume.

At 2 mg/L of SMX (Figure 6), the diffusion behavior is attributed to external diffusion and intraparticle diffusion in the pores. The constants ki and Ci are much higher than those obtained at 2µg/L. This shows that the parameters of Weber’s equation also depend on the solute concentration. The first segment corresponds to external diffusion (macropores and external particle surfaces), while the second segment is attributed to intraparticle diffusion in certain pores whose dimensions are likely to fall within the upper mesopore bracket (k_i2_ = 4.2 mg/g min^0.5^ and C_2_ = 50.3 mg/g and R^2^ = 0.99). A high SMX concentration apparently promotes the constriction of certain access routes and prevents micropore filling. The first phase lasted 107 min and the second 162 min. The rise in the initial solute concentration triggers an increase in the solute concentration in the boundary layers and hence, the thickness of these layers. This rise in concentration augments the driving force for the concentration gradient, hence, in the increase in molecular diffusion at the adsorbent surfaces. The intraparticle diffusion rate constant ki1 is very high and the y-intercept of the external diffusion phase is negative. The passage of the intercept “C_1_” below zero and the increase in the diffusion coefficient with the increase in the concentration of SMX (2 mg/L) indicate (i) the effect of external film diffusion resistance and (ii) that the monolayer is exceeded and the system evolves towards multilayer. The surface diffusivity is probably divided into two regions: a monolayer region and a multilayer region.

The Weber and Morris model offers a good description of SMX sorption kinetics by the ACP Norit SA Super with coefficients of determination greater than 0.99. It shows that (i) external mass transfer and intraparticle diffusion curb the adsorption rate, (ii) the transport rate of SMX molecules is governed by intraparticle diffusion, and (iii) the type and order of adsorption kinetics are not uniform. The next step consisted of examining adsorption kinetics using the three previously mentioned models.

#### 2.4.3. Adsorption Kinetic Modeling

**Figure 7:**
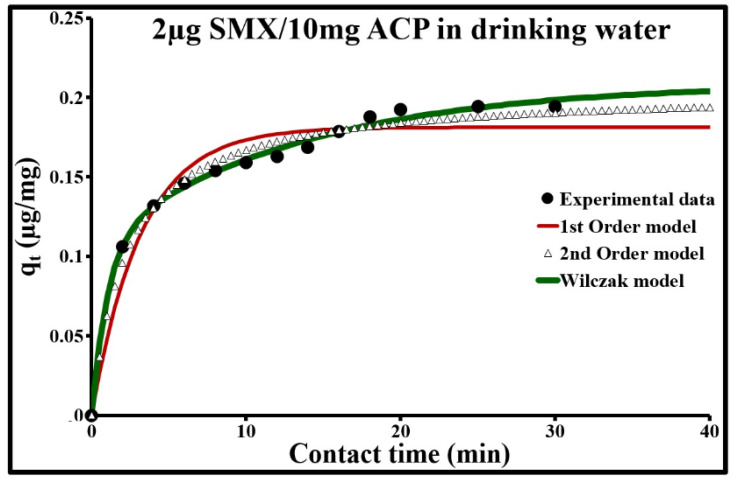
Comparison of adsorption kinetic models at 293 K for 2 µg/L of SMX and 10 mg/L of ACP Norit SA Super in drinking water.

**Figure 8:**
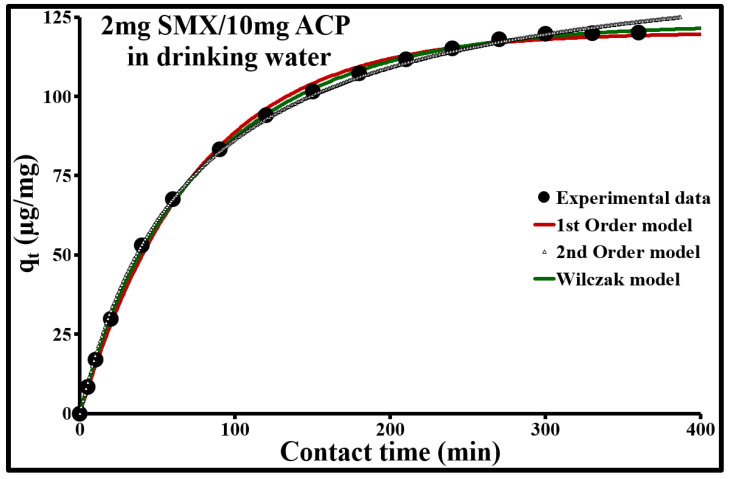
Comparison of adsorption kinetic models at 293 K for 2 mg/L of SMX and 10 mg/L of ACP Norit SA Super in drinking water.

**Figure 9:**
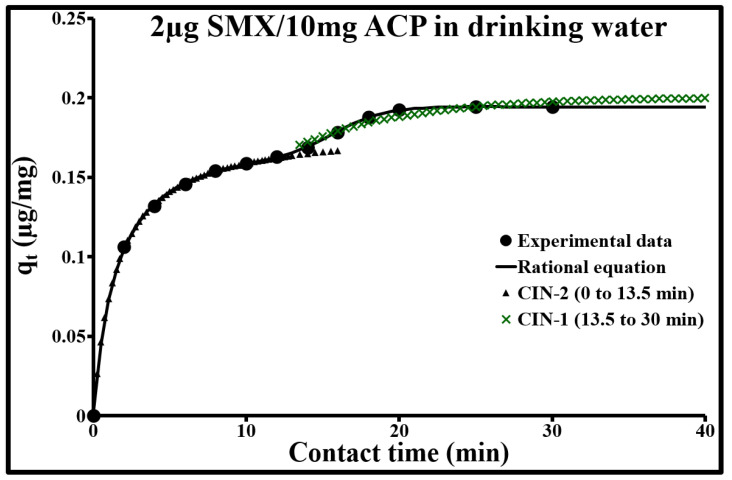
Modeling of the adsorption kinetics of 2 µg/L of SMX per part.

The comparison of experimental data with the three empirical models (Figure 7, Figure 8 and Figure 9) indicated, as an initial approximation, that the double exponential model applies fairly well to adsorption kinetics at both SMX concentrations. This model is characterized by a low ∆q and a calculated *q_e_* very close to the experimental *q_e_* (Table 4). More specifically, it includes two different adsorption rates, a rapid phenomenon and a second phase characterized by slower stabilization. These two phenomena can be represented, respectively, by a rapid external, or even internal, diffusion mechanism, and by a slow intraparticle diffusion phase. However, the sorption kinetics at an SMX concentration of 2 µg/L can be better modeled in steps (Table 5). From 0 to 13.5 min, it follows a second-order rate law with very rapid sorption, with 85% sorption in 13.5 min, and shows excellent agreement with the experimental values. During this phase, the pseudo-second-order model describes the involvement of both external mass transfer and intraparticle diffusion mechanisms. Above 13.5 min, the pseudo-first-order model applies well; it gives the best results with a low ∆q and a calculated *q_e_* equal to the experimental *q_e_*.

## 3. Materials and Methods

### 3.1. Materials

All reagents and solvents used were of analytical grades. The sulfamethoxazole used in this study (C_10_H_11_N_3_O_3_S, Sigma-Aldrich, Product Number S 7507, CAS number 723-46-6, 99.6% purity) was a white powder with a molecular weight of 253.28 g/mol, a boiling point close to 167.5 °C, an octanol/water partition coefficient of 0.89, and two acid dissociation constants defined by pK_a1_ ≈ 1.83 and pK_a2_ ≈ 5.57 [47]. Its geometric dimensions—length, height, and width—are 1.031, 0.587, and 0.526 nm, respectively; its Stokes diameter is about 0.76 nm (Nghiem et al. 2007); and its polarizability is between 150 and 170 a.u. The “Norit SA Super” powdered activated carbon (ACP) obtained from peat was supplied by JACOBI CARBONS (Cabot Norit Activated Carbon, Amersfoort, Netherlands). SMX and ACP were used as received.

### 3.2. Methods

#### 3.2.1. Characterization Techniques

The particle size distribution of ACP was obtained by helium-neon laser diffraction (632.8 nm), using the Malvern Mastersizer S particle size analyzer (Malvern Panalytical, Malvern, UK) by applying the Fraunhofer optical model. The specific surface areas, the pore volumes, and the slit widths less than 50 nm were determined at 77 K from nitrogen adsorption/desorption isotherms using a Micromeritics ASAP 2050 discontinuous volumetry sorptometer (Micromeritics Instrument Corp., Norcross, GA, USA). Prior to analysis, ACP was dried and degassed at 383 K until a residual vacuum of less than 0.02 mbar using the Micromeritics AccuPyc 1330 degassing ramp (Micromeritics Instrument Corp., Norcross, GA, USA). The BET method was used to determine the specific surface area. The t-plot method was used to assess the main characteristics of the micropores from the desorption isotherms. In addition to textural analysis, micropores smaller than 2 nm were characterized by CO_2_ adsorption at 273 K according to the Dubinin–Astakhove method [48]. The molecular cross-sectional areas of N_2_ and CO_2_ are 0.162 and 0.187 nm^2^, respectively. The point of zero charge (PZC) was determined using the Mular–Roberts [49] method, known as the pH drift method. This corresponds to the pH at which the total particle charge (including the surface of the mineral and the solvation shell layer) is zero. It can be representative of a complete absence of charge or of an exact balance of positive and negative charges present in the double layer. The activated carbon surface functional groups were identified and quantified by acid–base titration following the Boehm method [31,32,33]. Their determination was performed with NaHCO_3_ for carboxylic acid functions, with Na_2_CO_3_ for lactone and carboxylic acid functions, with NaOH for phenol, lactone, and carboxylic acid functions, and with HCl for basic functions. Excess base was back titrated with an HCl solution. The characterization of SMX was done by the analysis and processing of bibliographic data and also by its analysis in UV spectroscopy.

#### 3.2.2. Adsorption Method and Models

Kinetic and equilibrium adsorption were determined with batch experiments at 20 ± 1 °C. Experiments were conducted in drinking water. This water had a pH of 8.1 ± 0.1, a conductivity of 352 μS/cm at 25 °C, an ionic force of 0.0054 ± 0.0004 mol/L, and a chemical composition with a dissolved salt concentration of 259 ± 3 mg/L with 3.35 meq/L for cations and 3.51 meq/L for anions. SMX quantification was performed by liquid chromatography coupled to mass spectrometry for concentrations below 0.1 mg/L and by UV spectrometry for concentrations exceeding 0.1 mg/L. This value corresponds to the SMX quantification limit by UV spectrometry. The value of 0.1 mg/L was chosen as a reasonable limit for switching from UV spectrometry to chromatography. The limits of detection and quantification are 0.01 and 0.03 mg/L for UV spectrometry and 0.7 and 2 ng/L for chromatography. The solutions were prepared by dilution in amber high-density polyethylene bottles at ambient temperature.

For the realization of the adsorption isotherm, the adsorbent concentration was set at 10 mg/L and the SMX concentrations were at intervals ranging from 2 µg/L to 2 mg/L. A total of 10 mg/L of ACP corresponds to the optimal dose determined experimentally. Mixing, homogenization, and dispersion were performed using an overhead shaker at 60 rpm, for 4 h, which was found to be a sufficient time for an equilibrium to be attained. No stirrer was placed in the bottles (no magnetic stir bar). During this phase, a 40 mL sample from each bottle was centrifuged for 10 min at 20,379× *g*, then the supernatant was filtered with a polyvinylidene fluoride (PVDF) filter with an average porosity of 0.1 µm, then the SMX is determined. The adsorption isotherm was obtained by the graphic representation of the quantity of SMX adsorbed by the mineral “q_e_” according to the residual SMX concentration in the equilibrium solution “*C_e_*” [*q_e_* = f(*C_e_*)]. All experiments were carried out in a laboratory at a fixed temperature of 20 ± 1 °C (air-conditioned laboratory).

Given the texture and the energetic heterogeneity of the adsorbent surface, the adsorption isotherm models of Sips and Dubinin–Astakhov (D–A) were used to describe the adsorption equilibrium characteristics. The Sips model [50] is a combination of the Langmuir and Freundlich isotherm models, as shown in Equation (3).

(3) $$q_{e}=q_{\max}\frac{{\left(K_{s}C_{e}\right)}^{n_{s}}}{1+{\left(K_{s}C_{e}\right)}^{n_{s}}}$$

where *q_e_* is the amount of pollutant adsorbed per gram of the adsorbent at equilibrium (mg/g), *q_max_* is the maximum monolayer saturation capacity (mg/g), *C_e_* is the equilibrium concentration of adsorbate (mg/L), *K_s_* is the affinity constant (L/mg), and *n_s_* is the surface heterogeneity index (dimensionless), which varies from 0 to 1. When *n_s_* equals unity, the Sips isotherm returns to the Langmuir isotherm and predicts homogeneous adsorption. On the other hand, deviation of the *n_s_* value from the unity indicates a heterogeneous surface. In addition, when the denominator equals unity, the model resembles the Freundlich model.

The Dubinin–Astakhov model in Equation (4) does not assume that the surface is homogeneous or that the adsorption potential is constant, as is the case for the Langmuir model. Its theory of volume filling of micropores is based on the fact that the adsorption potential is variable and the enthalpy of adsorption is related to the degree of pore-filling. This isotherm assumes that the surface is heterogeneous and is expressed as follows:

(4) $$q_{e}=q_{\max}\exp\left[-{\left(\frac{\varepsilon }{E\sqrt{2}}\right)}^{n_{D}}\right]$$

(5) $$\varepsilon =RT\ln\left(1+\frac{1}{C_{e}}\right)=\Delta G$$

*q_e_* is the amount of pollutant adsorbed per gram of the adsorbent at equilibrium (mg/g), *q_max_* is the maximum monolayer saturation capacity (mg/g), *C_e_* is the equilibrium concentration of adsorbate (g/g), *ε* is the Polanyi potential, *E* is the average adsorption energy (kJ/mol), *n_D_* is the surface heterogeneity index, *R* is the universal gas constant (8.314 J/mol K), and *T* is the absolute temperature (K).

The average adsorption energy *E* was used for estimating the type of adsorption mechanism. For a magnitude of *E* between 8 and 16 kJ/mol, the adsorption process followed the chemical ion exchange, and values of E below 8 kJ/mol were characteristic of a physical adsorption process. When E > 16 kJ/mol, the adsorption is dominated by intraparticle diffusion. The value *n_D_* characterizes the distribution of the adsorption energy in the micropores and, indirectly, the distribution of micropore size. The heterogeneity of these two parameters increased as *n_D_* decreased from 2, and, conversely, their homogeneity increased for values above 2. The micropore size also decreased as *n_D_* increased [51].

Two adsorption kinetics were carried out. The first one was performed over 30 min with 2 µg/L of SMX and 10 mg/L of ACP and the second one over 360 min with 2 mg/L of SMX and 10 mg/L of ACP. The samples corresponding to the different time intervals were simply filtered and then, analyzed by HPLC. In order to study the sorption kinetics, two types of models were used. The first, a diffusion-controlled process, is the Weber and Morris intraparticle diffusion model Equation (6) [52]. The second, which assumes that the process is controlled by the adsorption reaction at the liquid/solid interface, combines the pseudo-first-order kinetic model Equation (7), the pseudo-second-order kinetic model Equation (8), and the double exponential kinetic model Equation (9). The double exponential kinetic model provides insight into the adsorption of a solute on two types of sites. This model includes two different adsorption rates, a rapid phenomenon and a second phase characterized by slower stabilization. These two phenomena can be represented, respectively, by a rapid external and/or internal diffusion mechanism, and by a slow intraparticle diffusion phase.

These models are described by the following equations:

(6) $$q_{t}=k_{i}\sqrt{t}+C$$

(7) $$q_{t}=q_{e}\left[1-exp\left(-k_{1}t\right)\right]$$

(8) $$q_{t}=\frac{k_{2}q_{e}^{2}t}{1+k_{2}q_{e}t}$$

(9) $$q_{t}=q_{0}+q_{r}\left[1-exp\left(-k_{s}t\right)\right]+q_{s}\left[1-exp\left(-k_{r}t\right)\right]$$

with *q_t_* the amount of adsorbed solute at time *t* (mg/g), *t* the time (min), *k_i_* is intraparticle diffusion rate constant (mg/g min^0.5^), *C* (mg/g) is a constant related to the thickness of the boundary layer, *q_e_* its value at equilibrium (mg/g), *k*_1_ the pseudo-first order rate constant (1/min), *k*_2_ the pseudo-second order kinetic rate constant (g/mg min), *q*_0_ is the amount of adsorbed solute at *t* = 0 min, *q_r_* and *q_s_* are the amount of adsorbed solute of the rapid and the slow step, respectively, and *k_s_* and *k_r_* are rate parameters (1/min).

These non-linear forms have been used as such to interpret the experimental results. In order to quantitatively compare the applicability of different kinetic models in fitting the data, a normalized standard deviation, Δq, was also calculated as follows [53]:

(10) $$∆q\left(\%\right)=100\sqrt{\frac{1}{n-1}\sum^{\;}{\left[\frac{q_{t,exp}-q_{t,cal}}{q_{t,exp}}\right]}^{2}}$$

where *n* is the number of data points; and *q_t,exp_* and *q_t,cal_* are the experimental values and the values calculated by the model, respectively.

The appropriate model to describe the adsorption kinetics of each system was determined based on the comparison of R^2^ and the normalized standard deviation Δ*q* (%). The determination of R^2^ alone is insufficient to decide among the kinetic models.

## 4. Conclusions

Surface chemistry, specific surface area, pore size distribution, and electrokinetic properties are important parameters governing the sorption mechanism and the quantity of SMX adsorbed by the activated carbon Norit SA Super. The quantities of SMX often found in polluted waters (≤2 µg/L) can be completely adsorbed by very small quantities of activated carbon (≤10 g/m^3^ of water). At tap water pH, SMX and ACP are both negative and their interaction is of a physical nature. The π–π dispersive interactions and electrostatic interactions produced by bridging using Ca^2+^ and/or Mg^2+^ are very likely to be predominant and to control adsorption.

Given the heterogeneity of the activated carbon’s surface energy due to its internal and external structure, sorption kinetics can be perfectly modeled first by a second-order rate law for the micropore sequence and then, by a first-order rate law for the mesopore and macropore sequence. The external and intraparticle diffusion phenomena are determining factors in the adsorption process. The intraparticle diffusion model clearly identifies all the phases involved. It also confirmed that the initial concentration affects the process kinetics. Modeling of the overall kinetics using a double exponential model is able to cover all these phases and gives a very significant mean with a very good coefficient of determination and low deviation between experimental data and calculated values.

This investigation into adsorption kinetics and mechanisms of a very small quantity of a pharmaceutical residue, which is persistent and mobile in soils, shows low biodegradability, and is frequently found in the different aquatic compartments, constitutes progress in both theoretical and practical aspects. From an industrial perspective, activated carbon offers rapid adsorption kinetics and is characterized by easy implementation and low sludge production (≤10 g/m^3^ of water). It can be used in different processes. The adsorption in tap water and examination of the adsorption kinetics of very reliable quantity of SMX with a very small quantity of ACP, the innovative element of this study, show that the results obtained are very encouraging for the use of ACP on an industrial scale for the removal of SMX. In terms of future prospects and prior to widespread application, the field of application would need to be extended to other pharmaceutical pollutants and semi-pilot industrial studies would have to be conducted.

## Figures and Tables

**Table 1:** Main characteristics of ACP Norit SA Super.

da µm	PZC	SS_BET_ m^2^/g	SS_µP-t_ m^2^/g	SS_mP-t_ m^2^/g	SS_MP-t_ m^2^/g	L_µ-t_ nm	V_µP-t_ cm^3^/g	V_mP-t_ cm^3^/g	V_MP-t_ cm^3^/g	SS_µP-DA *_ m^2^/g	V_µP-DA *_ cm^3^/g
24	6.8	957	695	203	60	1.75	0.30	0.17	0.10	711	0.31

da—average diameter. PZC—Point of zero charge. t—t-plot method. DA—Dubinin–Astakhov method. * Analysis done with CO_2_. SS—Specific area. SS_BET_—BET specific surface area. SS_µP_—Equivalent specific surface area of micropores. SS_mP_—Equivalent specific surface area of mesopores. SS_MP_—Equivalent specific surface area of macropores. L_µ-t_—Mean Equivalent pore width (t-Plot). V_µP_—Specific micropore volume. V_mP_—Specific mesopore volume. V_MP_—Specific macropore volume.

**Table 2:** Functional groups on activated carbon Norit SA Super by Boehm analysis.

Functional Group	Density µmol/m^2^	Number of Sites/nm^2^
Carboxylic	0.28	0.17
Carbonyl	0.46	0.28
Anhydride	0.17	0.10
Lactone	0.06	0.03
Phenol	0.53	0.32
Total electron donor	0.96	
Total electron acceptor	0.80	

**Table 3:** Kinetic parameters of Weber–Morris model.

		External Diffusion		Intraparticle Diffusion				
				Micropore Diffusion		Mesopore Diffusion		
C_ACP_ mg/L	C_0_ mg/L	k_i1_ mg/g min^0.5^	C_1_ mg/L	k_i2_ mg/g min^0.5^	C_2_ mg/g	k_i3_ mg/g min^0.5^	C_3_ mg/g	R^2^
10	0.002	0.039	0.052	0.014	0.113	0.038	0.027	0.99

**Table 4:** SMX adsorption kinetic parameters given for the three kinetic models.

		Pseudo-First Order				Pseudo-Second Order			
**C_0_** **mg/L**	**q_e.exp_** **mg/g**	**q_e_** **mg/g**	**k_1_** **min^−1^**	**R^2^**	**∆q** **%**	**q_e_** **mg/g**	**k_2_** **g/mg min**	**R^2^**	**∆q** **%**
0.002	0.198	0.181	0.313	0.948	8.8	0.205	2.146	0.985	4.6
2	120.5	120.16	0.013	0.999	4.4	147.9	9.5 × 10^−5^	0.999	1.9
**Double-Exponential Model**									
**C_0_** **mg/L**	**q_e.exp_** **mg/g**	**q_0_** **mg/g**	**q_e1_** **mg/g**	**q_e2_** **mg/g**	**q_e1_ + q_e2_** **mg/g**	**k_D1_** **min^−1^**	**k_D2_** **min^−1^**	**R^2^**	**∆q** **%**
0.002	0.198	1.5 × 10^−7^	0.102	0.107	0.209	0.075	0.979	0.996	1.9
2	120.5	1.5 × 10^−5^	107.7	15.0	122.7	0.011	0.049	0.999	2.5

**Table 5:** Kinetic parameters of stepwise modeling applied to the adsorption of 2 µg/L of SMX per 10 mg/L of activated carbon.

		0 to 13.5 min Pseudo-Second Order				13.5 to 30 min Pseudo-First Order			
C_0_ mg/L	q_e.exp_ mg/g	q_e_ mg/g	k_2_ g/mg min	R^2^	∆q %	q_e_ mg/g	k_1_ min^−1^	R^2^	∆q %
0.002	0.198	0.181	3.730	0.999	0.6	0.200	0.140	0.998	1.6
